# Kraft Process—Formation of Secoisolariciresinol Structures and Incorporation of Fatty Acids in Kraft Lignin

**DOI:** 10.1021/acs.jafc.1c00705

**Published:** 2021-05-18

**Authors:** Maarit H. Lahtinen, Joona Mikkilä, Kirsi S. Mikkonen, Ilkka Kilpeläinen

**Affiliations:** † Department of Food and Nutrition, University of Helsinki, P.O. Box 66, (Agnes Sjöbergin Katu 2), Helsinki FI-00014, Finland; ‡ Department of Chemistry, University of Helsinki, P.O. Box 55, Helsinki FI-00014, Finland; § Department of Microbiology, University of Helsinki, P.O. Box 56, Helsinki FI-00014, Finland; ∥ Helsinki Institute of Sustainability Science (HELSUS), University of Helsinki, P.O. Box 65, Helsinki FI-00014, Finland

**Keywords:** Kraft and residual lignin, mild thermal treatment, condensation reaction

## Abstract

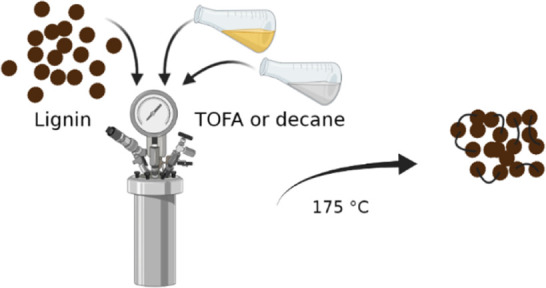

The complex chemical structure and the fact that many areas in pulping and lignin chemistry still remain unresolved are challenges associated with exploiting lignin. In this study, we address questions regarding the formation and chemical nature of the insoluble residual lignin, the presence of fatty acids in kraft lignin, and the origin of secoisolariciresinol structures. A mild thermal treatment of lignin at maximum kraft-cooking temperatures (∼170 °C) with tall oil fatty acids (TOFA) or in an inert solvent (decane) produced highly insoluble products. However, acetylation of these samples enabled detailed chemical characterization by nuclear magnetic resonance (NMR) spectroscopy. The results show that the secoisolariciresinol (β–β) structure in kraft lignin is formed by rearrangement of the β-aryl ether structure. Furthermore, fatty acids bind covalently to kraft lignin by reacting with the stilbene structures present. It is highly probable that these reactions also occur during kraft pulping, and this phenomenon has an impact on controlling the present kraft pulping process along with the development of new products from kraft lignin.

## Introduction

Material production from sustainable resources is an ongoing global challenge. Kraft pulping is currently the main processing technique to produce renewable cellulosic fibers used in various products.^1,2^ During the kraft pulping process, the most abundant linkage of lignin, namely, the β-O-4 linkage (45–50% in softwood),^1,2^ is mainly cleaved to form soluble products in the presence of hydroxyl and hydrogen sulfide anions. However, part of the β-O-4 linkages, 3–7% according to quantitative NMR experiments,^3,4^ remain intact during the process. The main side product formed from the kraft process, that is, sulfur-containing kraft lignin, is still usually burned as energy but could potentially be used to replace many applications based on scarce oil sources.

Kraft pulping delignification can be divided into three stages, namely initial, bulk, and residual delignification.^1^ After the fast bulk delignification stage, about 90–95% of the original lignin has been dissolved, depending on the processing conditions.^1,5^ The rate of delignification is much slower at the final residual delignification stage. Compared to the conventional kraft pulping process, processing parameters have been optimized, for example, by lowering the processing temperatures from 170 °C, and by tuning the concentrations of hydroxyl and hydrogen sulfide ions for more efficient delignification (i.e., extended cooking).^5^

Although the kraft lignin process is currently highly efficient, all aspects are not totally understood. Examples include the residual lignin in kraft pulping, which is highly insoluble and difficult to remove, and the occurrence of fatty acids in kraft lignin. From earlier studies, residual lignin is known to contain β-O-4 structures (3–7%) and “condensed aromatic structures”.^3,4,6−8^ According to the present view, the aromatic condensed structures are accumulated, rather than formed by chemical reactions during the process.^8^ Secoisolariciresinol is also one of the structures found in residual kraft lignin (2%), in addition to resinol (i.e., β–β, 2%) and phenyl coumaran (i.e., β-5, 5%).^9^ Processing conditions (conventional or extended cooking) affect the structure of residual lignin, in terms of the amount of phenolic groups, β-O-4 linkages, and condensed lignin units.^10^ Lignin–carbohydrate complexes have also been suggested to play some role in the more difficult delignification of residual lignin.^3,11,12^ Earlier studies by Gellerstedt et al. (1987, 2004) suggested that residual lignin contains also material resulting from the formation of aliphatic C–C bonds because corresponding signals appear in the NMR spectra of kraft lignins and insoluble material that remains in kraft lignin cooked with 2,6-xylenol after thioacidolysis.^9,13^ Furthermore, the involvement of radical reactions during the formation of residual lignin has been suggested, as fatty acids are incorporated into lignin during kraft pulping.^9^

The resinol structure is the major type β–β structure of lignin, whereas the secoisolariciresinol structure presents another, a minor type β–β structure. The secoisolariciresinol structure is probably not, as initially believed, formed through the resinol structure, that is, the more typical β–β structure in lignin.^14,15^ The amount of secoisolariciresinol structure in kraft lignin is 3%.^4^ Secoisolariciresinol is also one of the structures found in residual kraft lignin (2%).^9^ However, comparison of lignins in the same study showed that the amount of secoisolariciresinol structure, along with fatty acids, is higher in kraft and residual lignins as compared to milled wood lignin.^8^

In addition to the reactions occurring during kraft pulping, understanding the thermal reactions of lignins is important in the development of new processes based on pyrolysis and milder heat treatments.^16^ For example, kraft lignin has been suggested to be suitable for preparing pyrolysis oils or as a starting material for carbon fibers.^6,17,18^ Torrefaction is a milder thermal pretreatment for biomass taking place at 200–300, °C, which aims for improved fuel production or consumption.^19^ In all of these processes, lignin is thermally treated and information on the reactions involved is highly beneficial for their efficient development.

In order to investigate mild thermal heat treatments of lignin, the reactions were performed in tall oil fatty acids (TOFA) or in an inert solvent decane. Our initial aim was to study the reactions of kraft lignin and TOFA during mild thermal treatment at moderate temperatures (∼170 °C) to assess the incorporation of fatty acids into kraft lignin. The kraft lignin used was from the Lignoboost process, where lignin is precipitated using carbon dioxide (CO_2_) as the acid.^20^ TOFAs, obtained by distillation, were used in the reaction as a model compound representing the fatty acids released during the kraft pulping process. TOFA was also used as a reaction medium for heat transfer. The use of alkaline water solution containing hydrogen sulfide as the reaction medium was omitted, because the aim was to investigate radical reactions instead of ionic reactions. The material formed as a result of the heat treatment of lignin and fatty acids was hard, but very brittle, and furthermore dissolved very poorly in all of the tested organic solvents. According to initial chemical analysis, the material was composed of lignin and fatty acids, suggesting that the starting materials were attached through covalent bonding (it was impossible to “extract” any of the components with any solvents or their combinations). To understand the compositions of the materials and chemical reactions involved, a set of further experiments and analyses was performed. To define lignin–lignin reactions, a similar mild heat treatment was performed using the chemically inert decane instead of TOFA as the solvent. Chemical and thermal properties of the formed products were characterized thoroughly using various complementary techniques.

## Materials and Methods

### Materials and Reagents

Kraft softwood lignin (Metso, LignoBoost lignin), received in the powdered form, was dried further in an air-circulating oven at 40 °C before use. Distilled TOFA (product name: FOR2) was received from Forchem (Rauma, Finland), and typically comprises of certain rosin acids (total 2%), and the following fatty acids (total 96%) as the main components: linoleic acid (18:2, 42%), oleic acid (18:1, 32%), and pinolenic acid (18:3, 7%). Decane was purchased from Sigma-Aldrich (St. Louis, MO, USA). The solvent used for NMR (*d*_6_-dimethyl sulfoxide, DMSO-*d*_6_) was purchased from Eurisotop (Saint-Aubin, France).

### Procedure for Lignin Heat Treatment

To obtain solutions with 5 wt % of lignin with a similar level of volume, lignin (5.0 g in TOFA and 4.0 g in decane) and TOFA (95 g, *d* = 0.91 g mL^–1^) or decane (100 mL, *d* = 0.73 g mL^–1^) were placed in an inner PTFE cup of a 450 mL stainless-steel Parr reactor (Parr Instrument Company, Moline, IL, USA) equipped with a heating mantle, temperature control, and a pressure monitor (controller model 4843). The reactor was flushed, to remove air, by filling and emptying it three times with N_2_ (100 psi, 0.69 MPa). After flushing, N_2_ pressure was set at 70 psi (0.48 MPa), which was maintained during heating by releasing N_2_ through a valve. The reaction mixture was heated to 170 °C and maintained at that set temperature for 2 h. The product started to form already after 15 min, but to confirm that all lignin reacted, and for the purpose of comparison, a reaction time of 2 h was used. The monitored heating temperature was approximately 170–180 °C in decane and 170–195 °C in TOFA. After cooling, the floating reaction product (lignin–decane or lignin–TOFA) was collected from the surface of the liquid, washed with acetone, and dried in an air-circulation oven at 40 °C. Part of the product could not be easily removed from the temperature sensor of the Parr reactor, and therefore reliable mass balance calculations could not be performed.

### Acetylation of Samples for NMR Analysis and SEC

Starting lignin or the heat-treated sample (100–150 mg) was placed in a round bottom flask. Pyridine (2 mL) and acetic anhydride (2 mL) were added. The mixture was allowed to react at room temperature without stirring for 20 h, which resulted in dissolution of the starting lignin. The polymerized samples were further refluxed at 80 °C with stirring for 7 h, resulting in mainly soluble products. The acetic anhydride was quenched by adding and evaporating ethanol twice (20 mL), the trace of pyridine was evaporated with toluene (four times, 20 mL), and finally the trace of toluene was evaporated with chloroform (twice, 20 mL).

### Chemical Characterization by FT-IR Spectroscopy

The FT-IR spectra of the starting lignin, TOFA, lignin–decane, and lignin–TOFA were recorded with a Bruker ALPHA (Bruker Corporation, Billerica, MA, USA) attenuated total reflectance (ATR)-FT-IR spectrometer in a spectral range of 375–4000 cm^–1^.

### Thermogravimetric Analysis

Thermal decomposition was analyzed by thermogravimetric analysis (TGA) using a Mettler Toledo TGA/SDTA 851e (Mettler-Toledo International Inc.). The samples (5–10 mg) were analyzed in alumina crucibles with pinhole lids. A heating rate of 10 °C min^–1^ and an N_2_ flow rate of 50 mL min^–1^ were used.

### Differential Scanning Calorimetry

Differential scanning calorimetry (DSC) measurements were performed using a DSC Q200 (TA Instruments, NJ, USA). The samples (5–10 mg) were analyzed in aluminum pans with lids. A heat–cool–heat cycle was used with a heating rate of 10 °C min^–1^ and a cooling rate of 5 °C min^–1^, and the measurements were performed under nitrogen. The maximum temperature used was below the degradation temperature of the material to be analyzed. A second heating stage was used to analyze the data of samples containing lignin.

### Detailed Characterization of the Chemical Structure by NMR Spectroscopy

The starting lignin, lignin–decane, and lignin–TOFA were characterized using NMR spectroscopy. All samples were analyzed in DMSO-*d*_6_ as the solvent. The thermally treated acetylated samples were mainly soluble in DMSO-*d*_6_ (1.6 mL). After filtration through a cotton wool plug using a Pasteur pipette, the sample was concentrated under vacuum to a volume of 0.7 mL.

The 2D ^1^H–^13^C heteronuclear single-quantum coherence (HSQC) NMR spectrum of the starting lignin was acquired using a Bruker 600 MHz spectrometer, and the 2D HSQC NMR spectra of lignin–decane and lignin–TOFA using a Varian Unity Inova 600 MHz spectrometer. The temperature used during measurement was 25 °C for the starting lignin and 40 °C for heat-treated lignins. The pulse sequence used was hsqcetgpsisp2.2 (Bruker) or gHSQC (Varian). The 90° pulse width was determined separately for all samples. The number of increments used in the measurements was 256 (Varian) or 300 (Bruker), and the number of scans was 64 (Varian) or 512 (Bruker).

The TOFA used for the reaction was analyzed by ^1^H NMR before and after heat treatment. Spectra are presented in the Supporting Information. The fatty acid contents of the starting lignin and lignin–TOFA were evaluated from the ^1^H NMR spectra (Supporting Information) by comparing the integrals of fatty acid Fω (CH_3_, ∼0.9 ppm) and lignin Ar–OCH_3_ (∼3.8 ppm, including overlapping signals from the side-chain structures of lignin).

### Pyrolysis-Gas Chromatography/Mass Spectrometry

Measurements were performed using a Pyrolab2000 pyrolyzer connected to a Bruker Scion SQ 456 GC–MS. The pyrolysis chamber temperature was kept at 150 °C and the samples were pyrolyzed by heating the platinum filament in 8 ms to 200 °C and keeping the temperature elevated for 2 s before rapid cooling of the filament. The second heating to 580 °C was also 2 s long. Helium was used as a carrier gas at a flow rate of 1 mL min^–1^. Injector temperature was 250 °C and a 1:20 split ratio was used. Pyrolysis products were separated in an Agilent DB-5MS UI [(5%-phenyl)-methylpolysiloxane, 30 m × 0.250 mm × 0.25 μm film] capillary column. Column oven temperature was kept at 50 °C for 2 min after which the temperature was increased at a rate of 10 °C min^–1^ to 280 °C and kept at 280 °C for 5 min resulting in a 30 min overall run time. Ion source temperature was 250 °C with an electron ionization of 70 eV. The MS scan range was 40–400 *m*/*z*. Compounds were identified by comparing them to the National Institute of Standards and Technology (NIST) database.

### Analysis of Molar Masses by SEC

Molar masses of acetylated lignin–TOFA and starting lignin acetylated in the presence of TOFA (roughly 50 wt %) were analyzed using SEC. The Waters Acquity APC system was used for the analysis (Waters Corporation, Milford, MA, USA), equipped with Acquity APC XT columns 45, 200, and 450 Å, and using 30 °C as the column oven temperature. Sample concentration was 1 mg mL^–1^ in tetrahydrofuran (THF). Samples were filtered through a 0.2 μm syringe filter (GHP Acrodisc 13 mm, Pall Corp., Ann Arbor, MI, USA) before injection (50 μL). Total run time was 12 min, with a flow rate of 0.8 mL min^–1^. Molar masses were evaluated using data from the UV range (254 nm), and the following polystyrene standards were used for calibration: 474, 840, 2500, 5010, 13,300, 32,300, 43,400, 76,000, and 139,400 Da (Polystyrene Calibration Kit, Scientific Polymer Products, Inc.) along with 321,000 and 526,000 Da (Fluka).

## Results and Discussion

The starting kraft lignin was heat-treated in decane or TOFA to discriminate the internal reactions of lignin from cross-reactions of lignin with TOFA. The treatments were performed at ca. 175 °C, which is the maximum temperature during the conventional kraft pulping process.^1^ The heat treatment in decane provided information on thermal reactions of kraft lignin alone in the selected temperature, whereas the participation of fatty acids in reactions was evaluated in TOFA. The first difference between treatments was observed when monitoring the reaction temperature: the temperature set at 170 °C remained fairly constant in decane (170–180 °C), whereas it increased spontaneously (170–195 °C) in TOFA, indicating that exothermic reactions were occurring in the reaction vessel.

### Structural Characterization of Lignin Before and After Heating in Decane (Lignin–Decane) or TOFA (Lignin–TOFA) by NMR Spectroscopy

To gain insights into the reactions occurring under thermal treatments, the chemical structures of the starting and heat-treated lignins were analyzed using nuclear magnetic resonance (NMR) spectroscopy. The samples were acetylated because the very low solubility of nonacetylated samples prevented analysis. The underivatized products were not soluble in many of the typical organic solvents tested, and only slightly soluble in aqueous NaOH-solutions. The main part of the acetylated products was soluble in the NMR solvent used (DMSO-*d*_6_), and it therefore should be kept in mind that the minor insoluble fraction may have some structural differences compared to the observed results.

In practice, all of the lignin was floating on the surface of the liquid used for reaction media after the reaction in TOFA or decane. However, part of the material was tightly attached to the temperature sensor of the reaction vessel jeopardizing determination of accurate mass fractions. To confirm that lignin was not dissolved during reaction, the used TOFA was analyzed before and after the reaction by ^1^H NMR analysis (Supporting Information). According to visual inspection, decane did not contain lignin-based materials.

**Figure 1:**
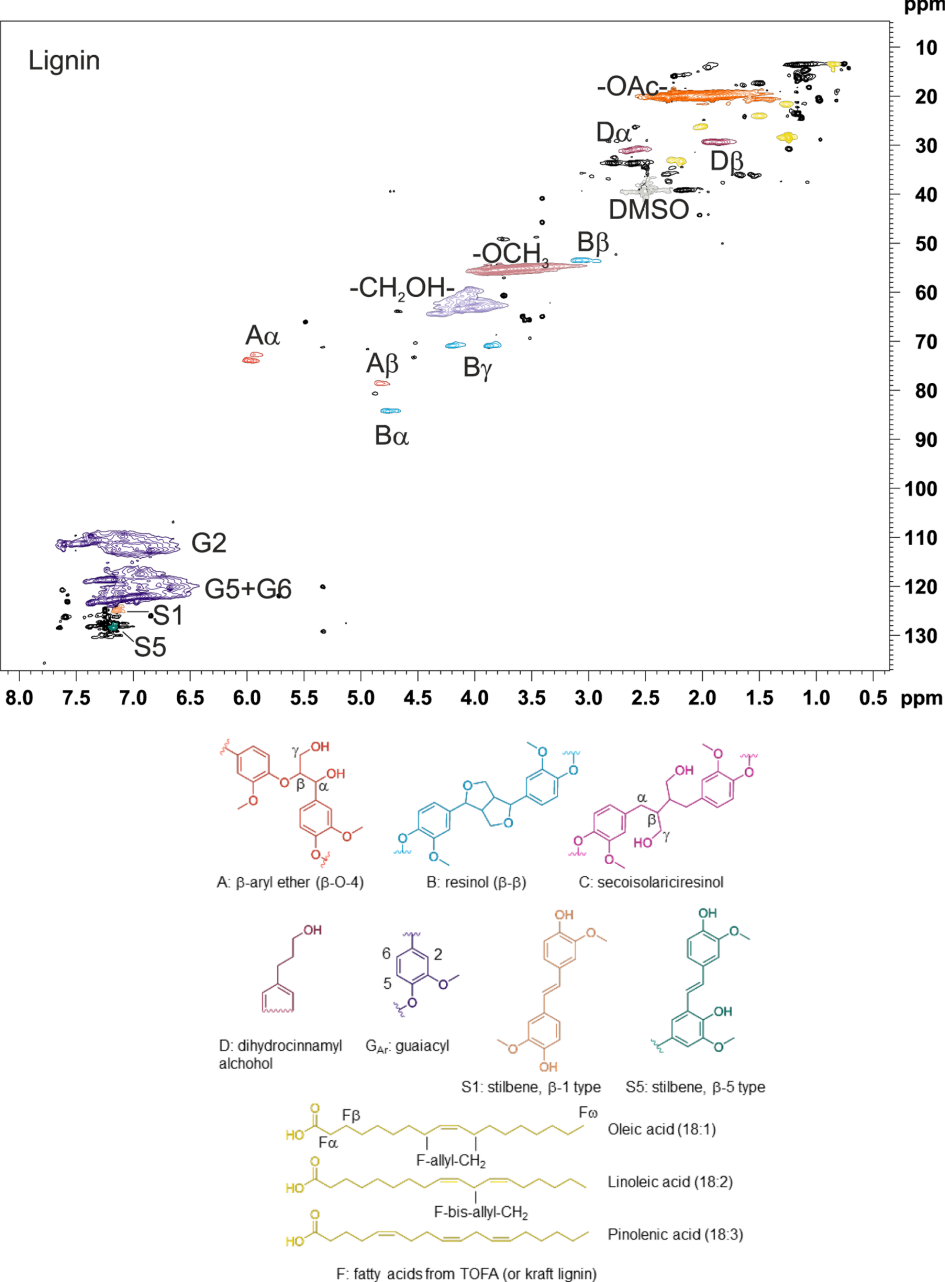
2D HSQC NMR spectrum of starting lignin (acetylated) and identified chemical structures.

**Figure 2:**
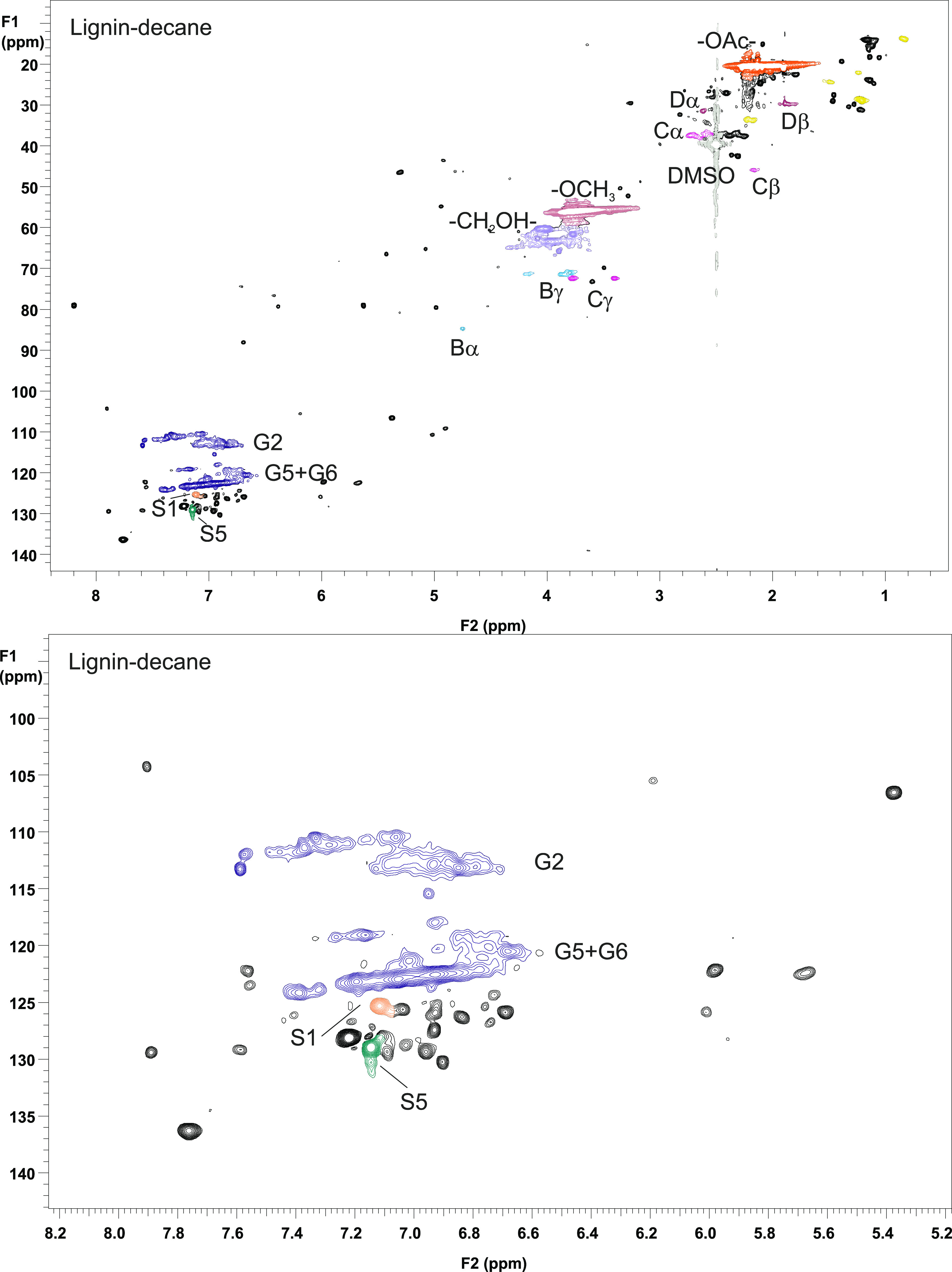
2D HSQC NMR spectrum of lignin heated in decane (top), and expansion of the aromatic area (bottom).

**Figure 3:**
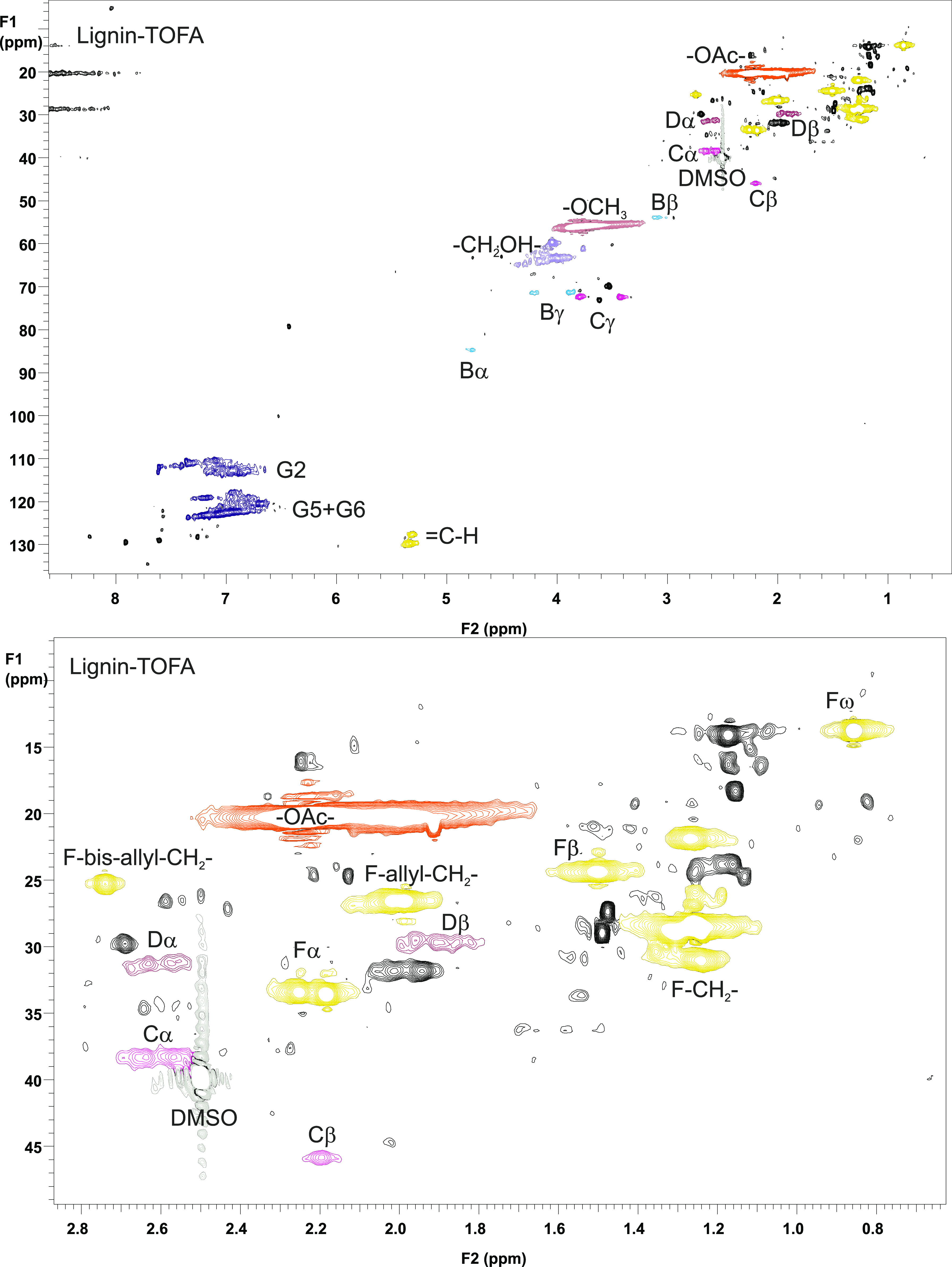
2D HSQC NMR spectrum of lignin heated in TOFA (top), and expansion of the aliphatic area.

The 2D HSQC NMR spectrum of the starting lignin and the structures identified in NMR spectra are presented in Figure 1. The 2D HSQC NMR spectrum of lignin–decane, with expansion of the aromatic area, is presented in Figure 2 and the spectrum of lignin–TOFA, with expansion of the aliphatic area, is presented in Figure 3. The NMR assignments were based on the previously published data: acetylated lignin samples,^21,22^ structures of acetylated secoisolariciresinol and non-acetylated dihydrocinnamyl alcohol,^14,23^ stilbene structures formed from phenylcoumaran (β-5) and spirodienone structures (β-1),^24^ and fatty acids of various chain lengths and degrees of unsaturation.^25^ It is worth noting that some of the published data are for nonacetylated samples and, therefore, some of the values may differ compared to acetylated samples used here. Furthermore, there might be some variation as a result of different solvents used for NMR analysis. Lists of identified cross peaks are presented in Table 1 for fatty acid-derived structures and in Table 2 for lignin-derived structures. From the cross-coupling patterns, the starting lignin (Figure 1) was composed mainly of β-aryl ether type (A) and resinol type (B) structures. The dihydrocinnamyl alcohol structure (D) was also present along with stilbene-type structures (S1 and S5). Some signals originating from fatty acids (F) were detected and those have also been observed earlier in samples of kraft lignin.^8,9,24^ As a rough estimation based on the ^1^H NMR spectrum, the fatty acid content of starting lignin was around 4 mol % (Supporting Information).

**Table 1:** Assignments of 2D HSQC NMR Spectra of Acetylated Starting Kraft Lignin, Lignin Heated in Decane (Lignin–Decane), and Lignin Heated in TOFA (Lignin–TOFA) in DMSO-*d*_6_ (fatty-acid derived structures)

	δ*H*/δ*C* (ppm)		
assignment/sample	lignin	lignin–decane	lignin–TOFA
signals from fatty acid-derived structures			
F–CH_2_–	1.26/21.72	1.24/21.97	1.27/21.54
F–CH_2_–	1.24/28.65	1.21/28.64	1.25/28.16
F–CH_2_–			1.24/30.71
Fω–CH_3_	0.85/13.51	0.83/13.71	0.86/13.45
Fβ–CH_2_–	1.49/24.14	1.49/24.23	1.50/24.03
Fα–CH_2_–	2.18/33.48	2.17/33.62	2.18/33.26
Fα–CH_2_^′^–	2.26/33.16	2.22/33.44	2.25/33.10
F-allyl–CH_2_–	1.99/26.23		1.99; 2.01/26.22
F-bisallyl–CH_2_–	2.74/24.90		
=C–H (F)			5.31/127.3
=C–H (F)			5.33/129.35

**Table 2:** Assignments of 2D HSQC NMR Spectra of Acetylated Starting Kraft Lignin, Lignin Heated in Decane (Lignin–Decane), and Lignin Heated in TOFA (Lignin–TOFA) in DMSO-*d*_6_ (lignin-derived structure)

	δ*H*/δ*C* (ppm)		
assignment/sample	lignin	lignin–decane	lignin—TOFA
**signals fromlignin-derivedstructures**			
β-aryl ether (A) α	5.99/74.08		
β-aryl ether (A) β	4.83/78.75		
resinol (B) α	4.76/84.39	4.77/84.64	4.77/84.38
resinol (B) β	3.09/53.62	?	3.08/53.44
resinol (B) γ	3.87/71.02	3.87/71.30	3.89/70.97
resinol (B) γ’	4.19/71.02	4.16/71.28	4.23/70.97
secoisolariciresinol α (C)		overlapping	2.57/37.98
secoisolariciresinol α’ (C)		2.73/37.31	2.67/37.96
secoisolariciresinol β (C)		2.16/45.82	2.20/45.51
secoisolariciresinol γ (C)		3.40/72.35	3.43/72.00
secoisolariciresinol γ’ (C)		3.78/72.38	3.79/71.98
dihydrocinnamyl alcohol (D) α	2.57/30.90	2.61/31.37	2.57/30.91
dihydrocinnamyl alcohol (D) α′	2.63/31.07	2.61/31.37	2.63/30.94
dihydrocinnamyl alcohol (D) β	1.85/29.45	1.86/29.51	1.91/29.36
dihydrocinnamyl alcohol (D) β′	1.90/29.45	1.88/29.69	1.96/29.19
stilbene, β-1 type α (S1)	7.14/124.99	7.12/125.33	
stilbene, β-5 type α (S5)	7.17/128.53	7.14/129.02	

Based on the NMR spectra of both lignin–decane (Figure 2) and lignin–TOFA (Figure 3), all the β-aryl ether type (A) linkages disappeared during heat treatment and secoisolariciresinol structures (C) were formed. The stilbene structures (S1 and S5) of lignin were unreactive in the inert solvent decane, but new, unknown signals appeared in the same area (125–130 ppm), indicating the possible formation of new unsaturated structures (Figure 2, expansion). The signals originating from stilbenes (S1 and S5) disappeared also in lignin–TOFA case, while the intensities and number of signals representing fatty acids (F) clearly increased (Figure 3, and the expansion). According to an estimation based on the ^1^H NMR spectrum, the fatty acid content of lignin–TOFA was around 7 mol %, almost twice as much compared to starting lignin (Supporting Information).

According to the results from the NMR analysis presented above, two sites seem reactive during heat treatment, also depending on the reaction media. In decane, β-aryl ether structures (A) are reacting but stilbenes (S1 and S5) are not, whereas both the β-aryl ether structures (A) and stilbenes (S1 and S5) react in TOFA. The formation of secoisolariciresinol (C) seems to be associated with reactions of β-aryl ether (A) because secoisolariciresinol (C) is formed and β-aryl ether (A) is reacting in both heat treatments, that is, decane and TOFA. The study by Zhang et al. 2003 concluded that secoisolariciresinol (C) is not formed from resinol (B) but instead is somehow connected to the β-aryl ether structure.^14^ Our findings support the earlier conclusions, but instead of a separate pathway in lignin biosynthesis, secoisolairciresinol (C) may form directly from β-aryl ether (A).

Stilbenes (S1 and S5) also react during heat treatment in TOFA, whereas these structures are stable in decane. Stilbenes and fatty acids, therefore, possibly form certain adducts or condensation products, although the site of connection could not be identified from the NMR spectrum of lignin–TOFA. The reaction pathway must be clearly more complex as more reacting components are present. The very obvious hypothetical product between lignin and TOFA, formation of an ester in a reaction with fatty acid and lignin could not be observed neither, in alignment with previous NMR work with kraft lignin, which contained fatty acids.^24^ Apparently, another experimental setup with some simpler model compounds is required for the identification of the formed products, which would be beyond the scope of the experiments presented in this paper.

### Chemical Characterization and Compositional Evaluation of Lignin–Decane and Lignin–TOFA by FT-IR Spectroscopy after Lignin Heat Treatment

**Figure 4:**
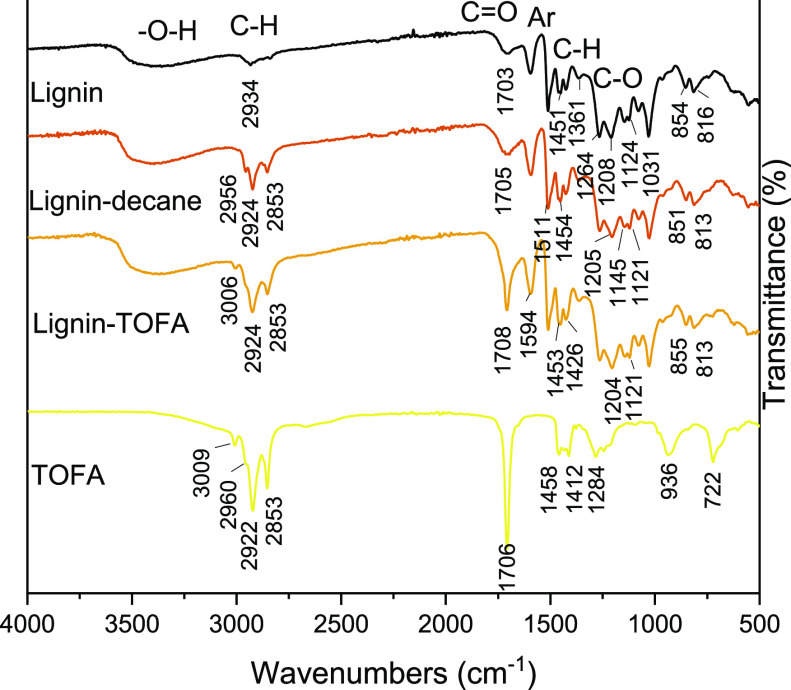
FT-IR transmission spectra of starting lignin and TOFA, and heat-treated lignin–decane and lignin–TOFA. Spectral range 500–4000 cm^–1^is shown. The spectra were obtained by means of ATR mode.

To further support the NMR analysis findings and to evaluate the compositions of the materials, we analyzed starting lignin, TOFA, and lignin heated in TOFA (lignin–TOFA) or decane (lignin–decane) using FT-IR (Figure 4). A list of the assigned absorption bands is presented in the Supporting Information. The FT-IR assignments for lignin are based on the previously published results for lignin.^26^ The FT-IR assignments for TOFA, a mixture of fatty acids produced as side products of the forest industry, are based on earlier studies on vegetable oils.^27,28^

The lignin samples were measured in earlier studies with KBr,^26,29,30^ and the values measured in this work, using FT-IR with ATR accessory with no need for separate sample preparation, may therefore vary slightly. For example, the spectrum of starting lignin has a weak absorption band at 1361 cm^–1^, which can be assigned to the aliphatic C–H stretch in methyl (not in −OMe) and to the phenolic hydroxyl group. Although the value slightly differs from the literature (1365–1370 cm^–1^) the position and shape of the absorption band are comparable.^26^ Similarly, the absorption band of lignin at a value of 1208 cm^–1^ may be assigned arising from C–C plus C–O plus C=O stretching (*G* condensed > *G* etherified), although the literature value is 1221–1230 cm^–1^).^26^

As seen in Figure 4, both heat-treated samples (lignin–decane and lignin–TOFA) have similar broad absorption bands for −OH stretching as the starting lignin (3500–3000 cm^–1^). Similarly, all the typical absorption bands found in the starting lignin, from absorption band 1703 cm^–1^ (C=O group) to 816 cm^–1^ (C–H out-of-plane in positions 2, 5, and 6 of *G* units), are also found in the heat-treated samples. These results also confirm that the material after heat treatment comprises of lignin, rather than totally charred components.

In Figure 4, the absorption band of the C=O group for lignin-decane (1705 cm^–1^) has a similar strength compared to starting lignin, whereas the strength of the C=O absorption band (1708 cm^–1^) for lignin–TOFA is similar compared to TOFA. Furthermore, the absorption band for C–H stretching in methine (3006 cm^–1^) is present in the spectrum of the lignin–TOFA sample, providing further proof that lignin–TOFA contains a notable amount of additional fatty acids.

For both heat-treated samples, the absorption bands at the area corresponding to either methyl or methylene C–H stretching (2960–2853 cm^–1^) were more intense compared to starting lignin, which would also be compatible with the formation of the secoisolariciresinol (C) structure with a relatively increasing amount of −CH_2_–.

### Thermal Characterization of Heat-Treated Lignin–TOFA by TGA and DSC

**Figure 5:**
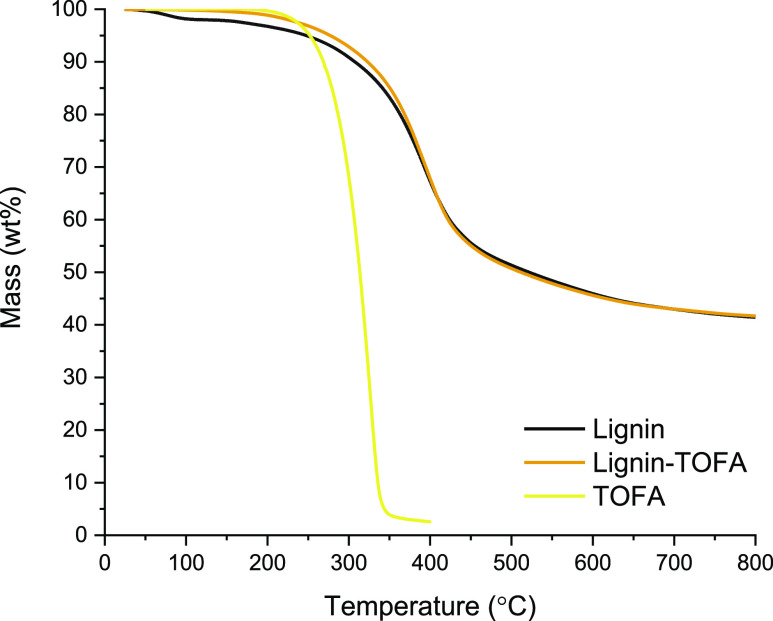
Thermogravimetric analysis of starting lignin, heat-treated lignin–TOFA, and TOFA.

To answer the question raised during the chemical analysis, that is, whether TOFA adsorbed into the lignin material during heat treatment in a way that would prevent washing of the unreacted fatty acids, thermal gravimetric analyses were performed on starting lignin, TOFA, and lignin–TOFA (Figure 5). Both samples containing lignin were clearly degraded and evaporated in a similar manner, and approximately 40 wt % of the material remained after heating to 800 °C. The mass loss of TOFA occurred at clearly lower temperatures, and less than 5 wt % of the material remained after heating to 400 °C.

**Figure 6:**
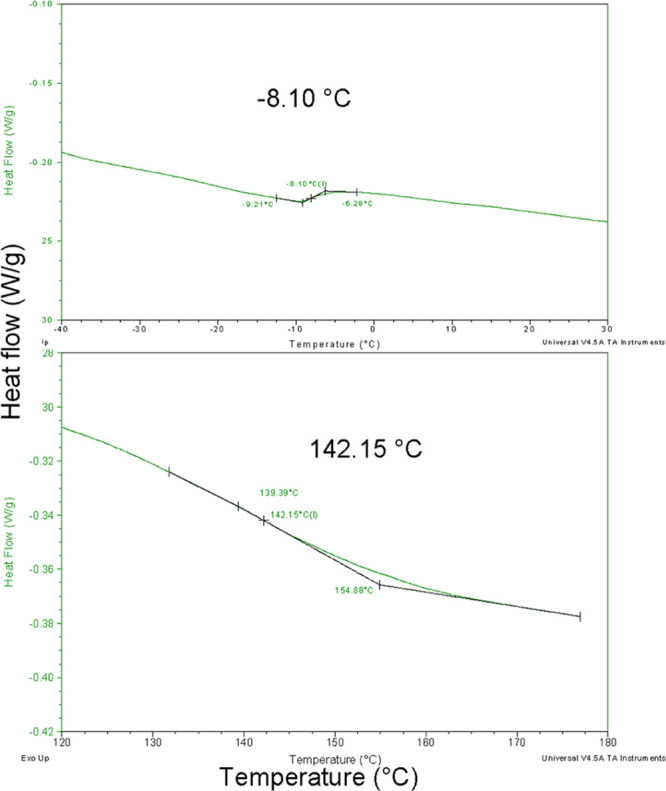
DSC curve of lignin–TOFA. For clarity, only the areas representing thermal changes are shown (the upper graph for *T*_m_ and lower for *T*_g_).

Thermal properties of starting lignin, TOFA, and heat-treated lignin–TOFA were also determined using DSC, presented for lignin–TOFA in Figure 6, and for starting lignin and TOFA in Supporting Information. According to our results, the glass transition temperature (*T*_g_) of the starting lignin was 156 °C, which is in the same range with previously reported values for kraft lignin (120–174 °C).^31,32^ For TOFA, transitions for crystallization and melting were observed at temperatures of −23 °C (*T*_c_) and −19 °C (*T*_m_), respectively. These values are reasonable compared to the DSC analysis of fatty acids in the literature, considering that the aim of the method used in this study was to compare the materials containing lignin and TOFA.^33^ The observed *T*_g_ was 142 °C for lignin–TOFA (Figure 6), which was slightly lower than that of the starting lignin. Another melting transition (*T*_m_) for lignin–TOFA was observed at −8 °C, which was in a similar range compared to transitions observed for TOFA.

The results from the thermal analyses also suggest that lignin and TOFA were connected by a covalent bond, because no separate evaporation of fatty acid was observed in the TGA of heat-treated lignin–TOFA, while this material contains some fatty acids according to chemical analyses (∼7 mol % by comparing ^1^H NMR integrals of lignin Ar–OCH_3_ and fatty acid Fω). Furthermore, the glass transition temperature of lignin–TOFA was slightly decreased compared to starting lignin and an additional melting transition was also observed.

### Pyrolysis-Gas Chromatography/Mass Spectrometry

Pyrolysis-gas chromatography/mass spectrometry (Pyr-GC/MS) was used to identify volatiles in order to understand the underlying mechanisms occurring during heat treatments. The Pyr-GC/MS analyses were performed by first heating the samples to 200 °C, mimicking the heat treatment performed earlier, and then bringing the temperature up to 580 °C. The results of the main peaks, each representing more than 3% peak area of the total amount of all peaks (100%), except for silylated compounds most probably originating from other sources, are shown in Table 3. All results are presented in the Supporting Information.

**Table 3:** Results of Pyr-GC/MS of Lignin and a Mixture of Lignin and TOFA^a^

pyrolysis temperature	200 °C		580 °C	
identification/sample	lignin	lignin–TOFA	lignin	lignin–TOFA
hexanal		4.24		
2,4-decadienal		17.20		
2-ethyl-2-hexen-1-ol		3.75		
(*E*)-2-decen-1-ol		5.78		
dimethyl disulfide	31.08	5.13		
guaiacol	36.71	5.13	13.35	14.07
4-methylguaiacol			27.85	29.14
4-ethylguaiacol			7.70	7.66
4-vinylguaiacol			14.08	15.03
eugenol			2.88	3.02
vanillin		17.49	2.26	2.08
isoeugenol		0.34	8.38	9.35
4-propylguaiacol		1.94	4.78	4.78

a First pyrolysis was performed at 200 °C and the second one at 580 °C. Results are presented as peak areas (%) of the total area of all peaks. Results for peaks more than 3% peak area are presented here; all results are found in the Supporting Information.

Pyrolysis at higher temperature (580 °C) produced a typical fragmentation pattern of kraft softwood lignin, and no significant difference was observed between the samples, lignin, and lignin–TOFA mixture. However, only two products, that is, dimethyl disulfide and guaiacol, were formed from the starting lignin at lower pyrolysis temperature (200 °C). In the lignin–TOFA mixture, vanillin was the main lignin-based compound released followed by guaiacol and dimethyl disulfide, and fatty acid-based 2,4-decadienal, 2-decen-1-ol, and hexanal were concurrently released.

**Scheme 1:**
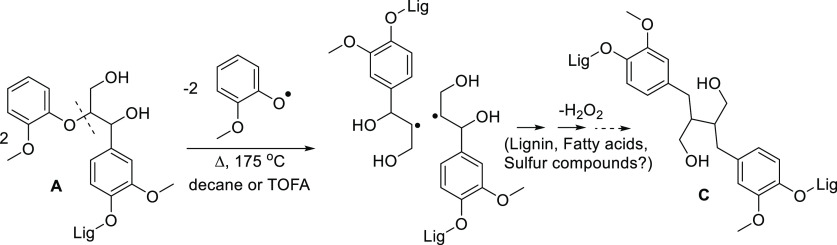
Proposed Reaction Mechanism for the Formation of Secoisolariciresinol (C) Structure from β-Aryl Ether (A)

The products formed from the pyrolysis of starting lignin were consistent compared to previous studies in both applied temperatures.^6,34,35^ The significant release of guaiacol at lower pyrolysis temperatures (200–300 °C) has also been observed earlier.^6,35^ Based on the structural analysis by the NMR presented above, the release of guaiacol also supports the hypothesis that secoisolariciresinol (C) is formed from β-aryl ether (A). Secoisolariciresinol (C) has previously been suggested to form during the biosynthesis of lignin from two coniferyl alcohol radicals and this structure would then be attached to the β-aryl ether structure (A).^14^ Previously, (C) has also been observed in DFRC (derivatization followed by reductive cleavage) analysis of lignin dimers, resulting from the DFRC procedure, in which β-aryl ether bonds are cleaved.^15^ Our results suggest that the Cβ–O bond of β-aryl ether structure is homolytically cleaved at mildly elevated temperatures (ca. 170–200 °C) or in other suitable conditions for prompting radical reactions, and the resulting Cβ-radicals couple to form the β–β bond (Scheme 1). The formation of the β–β bond would thus be similar to the suggested coupling of two coniferyl alcohol radicals.^14^ The formation of the secoisolariciresinol (C) structure then takes place after release of H_2_O_2_, which can react with either lignin or TOFA. The mechanism for the release H_2_O_2_ is not clear at this point, but the required reducing component could be present in a reaction media containing lignin, fatty acids, and sulfur compounds. Therefore, (C) formation does probably not occur during lignin biosynthesis from coniferyl alcohol, as suggested previously,^14,36^ but instead, the rearrangement of β-aryl ether, prompted by radical formation, may create a more chemically resistant, and possibly more insoluble structure.

The presence of TOFA clearly affected pyrolysis at 200 °C, but evaluation of plausible reaction pathways involved is more complex because of the higher number of reacting components. The similar formation of secoisolariciresinol (C), as suggested above, would be reasonable, because guaiacol was also released in the presence of TOFA. However, vanillin was the major lignin-based component released, suggesting cleavage of the Cα–Cβ bond. Results from the NMR analysis showed that stilbenes were concurrently consumed (disappearing). The formation of aldehydes from stilbenes is a known reaction under certain conditions, but based on the results presented here, it seems to require the presence of fatty acids, which also attach to lignin. The reaction pathway leading to the covalent bonding of TOFA under heat treatment is thus not evident from the present results, and requires further investigation.

### Analysis of Molar Masses of Starting Lignin and Lignin–TOFA

The molar masses of acetylated starting lignin and heat-treated lignin–TOFA were evaluated by SEC. The lignin sample was acetylated in the presence of TOFA for improved comparison of the samples (the SEC chromatograms can be found in Supporting Information). By visual inspection, the acetylated samples seemed to be soluble in the used solvent (THF). According to the results, presented in Table 4 for the signal with the largest area (i.e., the first signal of the chromatogram, see Supporting Information), the molar masses before and after heat treatment were very similar, and in fact, the molar mass of lignin–TOFA was slightly lower. Based on the low solubility of the product, this initially seemed surprising. However, this is well in line with the reaction pathway suggested for the formation of a secoisolariciresinol structure (C). This result is also consistent with the results of Balakshin et al. (2003), in that aromatic condensation products, that is, formed via coupling to the 5-position, are not produced during kraft pulping.^8^ Instead, cleavage of the C–O bond and rearrangement of β-aryl ether structure and formation of a new aliphatic C–C bond would result in a more hydrophobic and chemically resistant structure (Scheme 1). Notably, comparison of empirical formulas of two β-radicals formed from β-aryl ether (2 × C_10_H_12_O_4_H = C_20_H_24_O_8_H_2_) and the secoisolariciresinol dimer (C_20_H_22_O_6_H_2_) show that the relative reduction (−9%) in molar masses (from 394 to 360) is very similar compared to the molar masses acquired from SEC.

**Table 4:** Molar Mass Analysis of Starting Lignin and Lignin Heated in TOFA (Lignin–TOFA) Acquired Using SEC

molar mass/sample	lignin	lignin–TOFA
*M*_n_ (Da)	1800	1720
*M*_w_ (Da)	2570	2390
*M*_z_ (Da)	4030	3550
PDI (*M*_w_/*M*_n_)	1.427	1.390

### Impact of Results on the Kraft Pulping Process and Development of New Products by Heat Treatment

Cleavage of the β-O-4 linkage by the action of hydroxyl and hydrogen sulfide ions is the major ionic reaction pathway during kraft delignification. In addition, according to the results presented here, minor radical reaction pathways are involved in the kraft process. The existence of fatty acids and secoisolariciresinol structures in the kraft and residual lignins is known from earlier studies, and a radical pathway leading to these products has been proposed previously.^9^ However, the structural patterns of lignin taking part in these radical reactions is novel information, in addition to the reaction mechanism for the formation of secoisolariciresinol structure.

In this study, we have shown that temperature of 170–175 °C is one of the essential reaction conditions that induce the formation of these products through a radical pathway. Considering this temperature with the fact that delignification improves by lowering the temperature of conventional kraft pulping process, the formation of these products could be one of the reasons for the slow residual delignification phase. Unless derivatized by acetylation using harsh conditions, the products are highly insoluble in any common solvents, and could, therefore, form a protective layer, which is not easily accessible by the pulping chemicals anymore. Furthermore, the formed C–C bonds are more stable, less reactive, and more hydrophobic compared to the functional structures reacting in the starting materials. Understanding all underlying mechanisms of the kraft delignification process, even the minor ones, is useful for improved control of the whole process.

In addition to kraft pulping process, it seems that radical reactions of lignin are also most probably taking place during all heat treatment processes of lignin, and knowledge of the possible reaction pathways is useful in developing possible applications for lignin. However, further studies, in addition to the kraft lignin, are required to evaluate if the rearrangement of β-O-4 linkage to secoisolarisiresinol is taking place with other types of lignins as well.

Concluding the results presented here, β-aryl ether structures of lignin can rearrange to form secoisolariciresinol structures during mild thermal heat treatment. In addition, the formed structure with new C–C bonds and less hydroxyl groups is chemically more stable and at least partially responsible for the observed lower solubility of the formed product. On the other hand, if fatty acids are present, the stilbene structures of lignin react and form covalent bonds with fatty acids. Whereas the rearrangement of β-aryl ethers to secoisolariciresinol is likely to occur through radical reactions, the reaction pathway with fatty acids remains unclear. Finally, the knowledge of the reacting sites under mild thermal treatment of kraft lignin is valuable in the development of new applications based on heat treatment, along with controlling and optimizing the existing kraft pulping process.

## Supporting Information Available

List of selected FT-IR assignments; Pyr-GC/MS results, pyrograms, and pictures of materials after heat treatment; ^1^H NMR spectra of acetylated starting lignin and acetylated mixture of lignin and TOFA without heat treatment, TOFA before and after heat treatment, and acetylated lignin–TOFA after heat treatment; DSC curves of lignin and TOFA; and SEC curves (PDF)
