# Correction to Kraft Process—Formation of Secoisolariciresinol Structures and Incorporation of Fatty Acids in Kraft Lignin

**DOI:** 10.1021/acs.jafc.2c02007

**Published:** 2022-04-26

**Authors:** Maarit H. Lahtinen, Joona Mikkilä, Kirsi S. Mikkonen, Ilkka Kilpeläinen

Anna V. Faleva from the Northern (Arctic) Federal University named after M.V. Lomonosov commented on our article, noting that our nuclear magnetic resonance (NMR) assignments for the secoisolariciresinol structure were differing significantly when compared to values presented in the existing literature. As previously described in the original article, we thought that the values for NMR shifts differed due to the use of different solvents during the analysis. However, although the effect of solvents is well-known, the literature about various lignin model compounds measured in different solvents does not have such a significant variation.^1^ Additionally, we have recently identified part of the signals for the secoisolariciresinol structure in the starting lignin sample. Therefore, we have corrected the assignments for the two-dimensional (2D) heteronuclear single-quantum correlation (HSQC) NMR spectrum, as shown in Figure 1 (Figure 1 in the original article), by adding the assignments for secoisolariciresinol (E) and changing the identification of compound C to divanillyltetrahydrofuran, as suggested by Dr. Faleva. Furthermore, because structure C has been shown to be formed from E,^2^ all other aspects of the original article remain correct. In addition to the original article, E is further transformed to divanillyltetrahydrofuran during heat treatment.

**Figure 1:**
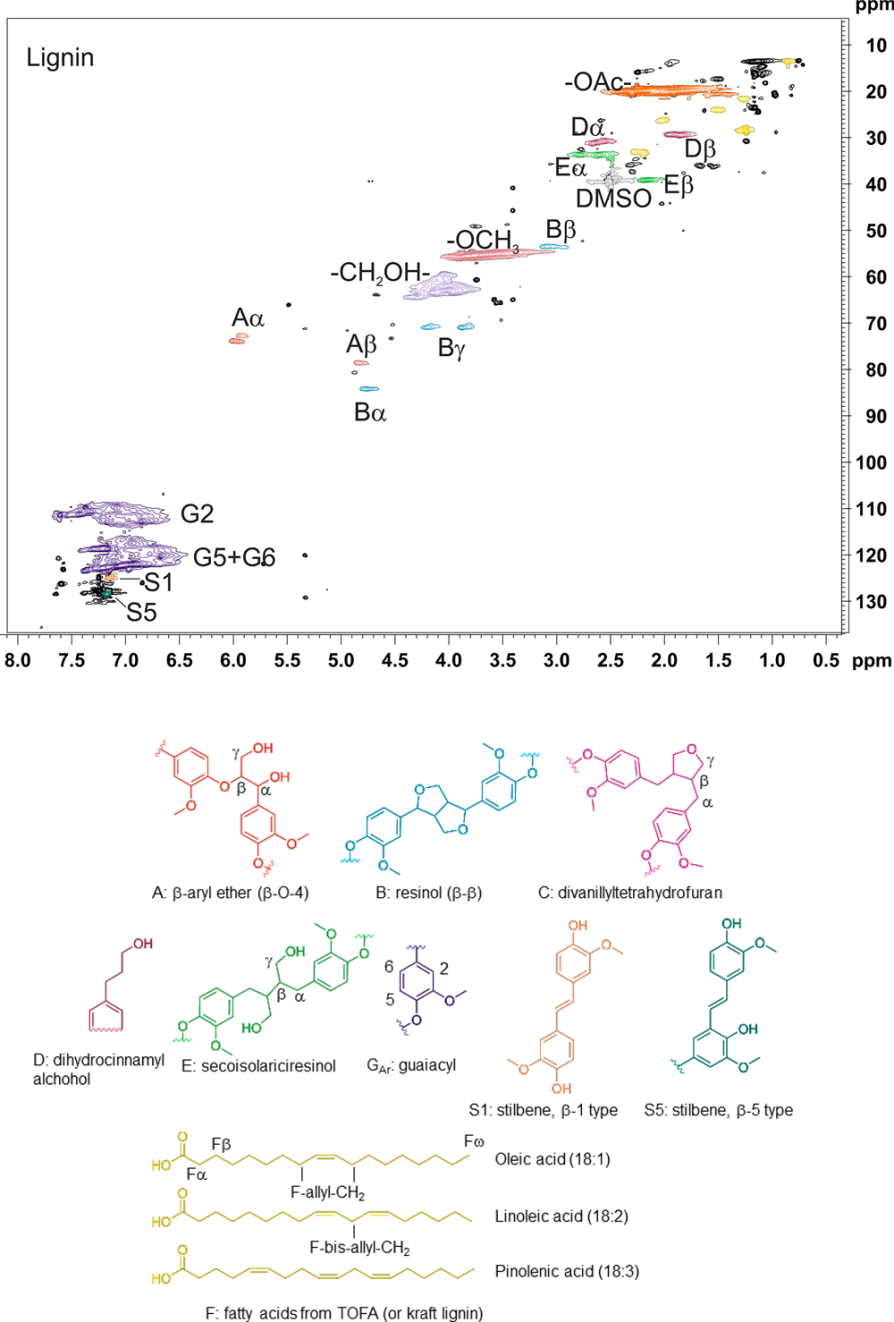
2D HSQC NMR spectrum of starting lignin (acetylated) and identified chemical structures. The sample was dissolved in DMSO-*d*_6_.

The NMR shifts reported earlier for the secoisolariciresinol structure were (acetylated, dissolved in acetone-*d*_6_) 2.64/35.2 and 2.78/35.2 ppm for α-CH_2_, 2.20/41.0 ppm for β-CH, and 4.01/64.8 and 4.20/64.8 ppm for γ-CH_2_.^3^ Our corrected assignments are very close to these values and indicate that they match [acetylated, dissolved in deuterated dimethyl sulfoxide (DMSO-*d*_6_)]: 2.61/33.8 and 2.78/33.8 ppm for α-CH_2_ and 2.15/39.3 ppm for β-CH, and γ-CH_2_ are overlapping with the other CH_2_OH signals.

In the original article, NMR shifts for the now newly identified structure C were in the case of the lignin–tall oil fatty acid (TOFA) sample (acetylated, DMSO-*d*_6_) 2.57/38.0 and 2.67/38.0 ppm for α-CH_2_, 2.20/45.1 ppm for β-CH, and 3.43/72.0 and 3.79/72.0 ppm for γ-CH_2_. NMR shifts for the model compound dimer found in the literature are very similar (non-acetylated, CDCl_3_): 2.52/39.2 and 2.58/39.2 ppm for α-CH_2_, 2.16/46.5 ppm for β-CH, and 3.56/73.3 and 3.91/73.3 ppm for γ-CH_2_.^4^ These assignments were further verified by 2D HSQC–total correlation spectroscopy (TOCSY) spectra (Figure 2).

**Figure 2:**
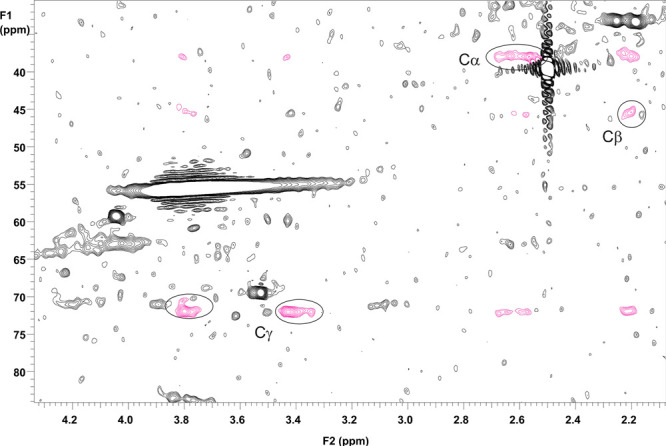
2D HSQC–TOCSY NMR spectrum of the lignin–TOFA sample (2D HSQC spectrum shown in the original article) showing the correlations of the divanillyltetrahydrofuran (C) lignin structure.
