# Supplementary material for: Kraft Process—Formation of Secoisolariciresinol Structures and Incorporation of Fatty Acids in Kraft Lignin
Source: J Agric Food Chem. 2021 May 18;69(21):5955–65. doi: 10.1021/acs.jafc.1c00705 (PMC8278485; doi:10.1021/acs.jafc.1c00705)
Supplement: Supplementary file 1 — jf1c00705_si_001.pdf [file jf1c00705_si_001.pdf]

Supporting Information for

Kraft process – formation of secoisolariciresinol structure and incorporation of fatty acids in kraft lignin

Maarit H. Lahtinen[†,‡*], *Joona Mikkilä*[‡,§], *Kirsi S. Mikkonen*[†,⊥], *Ilkka Kilpeläinen*[‡,⊥]

[†]Department of Food and Nutrition, P. O. Box 66, (Agnes Sjöbergin katu 2), FI-00014 University of Helsinki, Finland

[‡]Department of Chemistry, P. O. Box 55, FI-00014 University of Helsinki, Finland

[§]Department of Microbiology, P.O. Box 56, FI-00014 University of Helsinki, Finland

[⊥] Helsinki Institute of Sustainability Science (HELSUS), P.O. Box 65, FI-00014 University of Helsinki, Finland

Supporting Information Content:

Number of pages: 15

Number of tables: 2 (Table S1 and S2)

Number of figures: 14 (Figure S1 to S14)

Supporting information

Table S1. List of selected assignments of FT-IR absorption bands based on previously published data for lignin and vegetable oils

Table S2. Pyrolysis GC/MS, all results.

Figure S1. Materials after mild heat treatment at ~175 °C: lignin-decane, lignin-TOFA.

Figure S2. ^{1}H NMR spectrum of acetylated lignin before heat treatment. The integral value of 0.04 at ~0.9 ppm (Fω CH_3 of fatty acid) is related to the value of 1 at ~3.8 ppm (Ar-OCH_3 of a lignin unit and overlapping signals related to lignin side chain).

Figure S3. ^{1}H NMR spectrum of acetylated mixture of lignin and TOFA without heat treatment.

Figure S4. ^{1}H NMR spectrum of TOFA before heat treatment.

Figure S5. ^{1}H NMR spectrum of TOFA after heat treatment with lignin.

Figure S6. ^{1}H NMR spectrum of acetylated lignin-TOFA after heat treatment.

Figure S7. Differential scanning calorimetry (DSC) curve of starting kraft lignin.

Figure S8. DSC curve of tall oil fatty acids (TOFA).

Figure S9. Pyrogram of starting lignin from Pyr-GC/MS analysis at 200 °C.

Figure S10. Pyrogram of lignin-TOFA mixture from Pyr-GC/MS analysis at 200 °C.

Figure S11. Pyrogram of starting lignin from Pyr-GC/MS analysis at 580 °C.

Figure S12. Pyrogram of lignin-TOFA mixture from Pyr-GC/MS analysis at 580 °C.

Figure S13. Size exclusion chromatography (SEC) curve of starting kraft lignin.

Figure S14. Size exclusion chromatography (SEC) curve of lignin-TOFA.

Table S1. List of selected assignments of FT-IR absorption bands based on previously published data for lignin and vegetable oils (see the text for list of references). Abbreviations used for origin of signal: L = Lignin, F = Fatty Acid.

Observed maxima (cm-1)	Functional group and mode of vibration	Origin (L or F)
3000-3500	O-H stretching, phenolic and aliphatic hydroxyl (–OH)	L
2500-3300	O-H stretching, carboxylic acid (-COOH)	F
3009	C-H stretching, methine (=C-H)	F
2960	C-H asymmetric stretching, methyl ($-CH_3$)	F
2934	C-H stretching, methyl ($-CH_3$) and methylene ($-CH_2-$)	L
2922	C-H asymmetric stretching, methylene ($-CH_2-$)	F
2853	C-H symmetric stretching, methylene ($-CH_2-$)	F
1706	C=O stretching, carboxylic acid (-COOH)	F
1703	C=O stretching, conjugated aldehyde	L
1594	aromatic skeletal vibration	L
1511	aromatic skeletal vibration	L
1458	C-H bending, methyl ($-CH_3$) and methylene ($-CH_2-$)	F
1451	C-H bending, methyl ($-CH_3$) and methylene ($-CH_2-$)	L
1426	aromatic skeletal vibration combined with C-H in-plane bending	L
1412	=C-H bending, methine (=C-H)	F
1361	aliphatic C-H stretch, methyl ($-CH_3$, not in $–OCH_3$); an phenolic hydroxyl group	L
1284	C-O stretching	F
1264	guaiacyl ring plus C=O stretching	L
1208	C-C plus C-O plus C=O stretching (G condensed > G etherified)	L

1145	aromatic C-H in-plane bending, typical of G units (G condensed G etherified)	L
1124	secondary alcohols and C=O stretching	L
1031	aromatic C-H in-plane bending plus C-O bending in primary alcohols plus C=O stretching	L
936	C=C bending	F
854, 816	C-H out-of-plane in position 2, 5, and 6 of G units	L
722	methylene rocking $-(CH_2)_n-$, or/and C=C bending (*cis*)	F

Table S2. Pyrolysis GC/MS, all results. The left value in the cell is the %-area of the signal and the right value (in italic) is the retention time.

	Area of the signal (%) *Retention time (min)*			
Pyrolysis temperature	200 °C		580 °C	
Identification/Sample	Lignin	Lignin-TOFA	Lignin	Lignin-TOFA
Ethyl acetate	-	0.20 *2.96*	-	-
Dimethyl disulfide	31.08 *3.29*	5.13 *3.28*	-	-
Toluene	-	-	0.64 *3.56*	0.83 *3.57*
Dimethyl trisulfide	1.60 *7.01*	-	-	-
Hexanal	-	4.24 *4.05*	-	0.33 *4.04*
(4-Hydroxyphenyl)-phosponic acid	-	-	0.37 *7.19*	-
2-Pentylfuran	-	0.60 *7.25*	-	-
Decamethylcyclopentasiloxane	3.95 *8.84*	10.03 *8.95*	-	-
2-Methylphenol	-	-	0.81 *8.39*	-
Allyl benzyl ether	-	-	-	0.92 *8.40*
3-Methylphenol	-	-	-	0.86 *8.77*
p-Cresol	-	-	1.06 *8.76*	-
Guaiacol	36.71 *8.96*	5.13 *8.12*	13.35 *8.91*	14.07 *8.91*
(E)-4-Nonenal	-	1.44 *9.05*	-	-
(E)-2-Nonenal	-	0.63 *9.85*	-	-
2,4-Dimethylphenol	-	-	0.92 *9.90*	0.71 *9.91*
	-	-	-	0.40 *10.02*
6-Methylguaiacol	-	-	1.39 *10.33*	1.38 *10.33*
Cyclopentaneundecanoic acid	-	0.38 *10.08*	-	-
4-Methylguaiacol	-	-	27.85 *10.54*	29.14 *10.54*

Catechol	-	-	2.84 *10.76*	1.50 *10.79*
2,3-Dihydrobenzofuran	-	-	0.64 *11.03*	-
Trimethylsilyl 2-[(trimethyl-silyl)oxy]benzoate	1.31 *11.27*	-	-	-
(E)-2-Decenal	-	0.94 *11.47*	-	-
3-Methyl-1,2-benzenediol	-	-	1.84 *11.65*	1.32 *11.66*
4-Ethylguaiacol	-	-	7.70 *11.78*	7.66 *11.78*
Dodecamethylcyclohexasiloxane	8.86 *11.83*	3.47 *11.84*	-	-
2,4-Decadienal	-	17.20 *11.98*	-	-
4-Methyl-1,2-benzenediol	-	-	2.20 *12.10*	1.53 *12.13*
4-Vinylguaiacol	-	-	14.08 *12.33*	15.03 *12.33*
3-Methoxy-5-methylphenol	-	-	1.10 *12.46*	1.36 *12.47*
2-(Prop-2-enoyloxy)tetradecane	-	0.92 *12.75*	-	-
Dodecanoic acid, 2-(acetyloxy)-1-[(acetyloxy)methyl]ethyl ester	-	0.68 *12.82*	-	-
4-Ethyl-1,2-dimethoxybenzene	-	-	0.36 *12.84*	0.35 *12.84*
Eugenol	-	-	2.88 *12.89*	3.02 *12.89*
2-Ethyl-2-hexen-1-ol	-	3.75 *12.92*	-	-
(E)-2-Decen-1-ol	-	5.78 *12.96*	-	-
4-Ethenyl-1,2-dimethoxybenzene	-	-	0.78 *13.41*	0.76 *13.41*
Vanillin	-	17.49 *13.63*	2.26 *13.54*	2.08 *13.55*
5-Methoxy-2,3-dimethylphenol	-	-	0.37 *13.65*	0.42 *13.65*
3-Isopropoxy-1,1,1,7,7,7-hexamethyl-3,5,5-tris(trimethylsiloxy)tetrasiloxane	8.66 *14.13*	3.35 *14.13*	-	-
Isoeugenol	-	0.34 *14.21*	8.38 *14.16*	9.35 *14.16*
4-Propylguaiacol	-	1.94 *14.35*	4.78 *14.27*	4.78 *14.27*
Apocynin	-	1.20 *14.76*	1.44 *14.66*	1.37 *14.67*

2,5-Dimethoxyethylbenzene	-	-	0.37 *14.86*	0.44 *14.87*
1-(4-Hydroxy-3-methoxyphenyl)-2-propanone	-	0.68 *15.22*	0.78 *15.15*	0.50 *15.17*
4-((1E)-3-hydroxy-1-propenyl)-guaiacol	-	-	0.42 *15.74*	-
4-(3-hydroxy-1-propenyl)-guaiacol	-	-	0.40 *15.86*	-
Isopropyl pentakis(trimethylsilyl) disilicate	5.49 *16.11*	2.30 *16.11*	-	-
8-Hexadecyne	-	0.36 *16.75*	-	-
Decamethyltetrasiloxane	-	0.85 *17.80*	-	-
Bis(2-trimethylsilylethyl ester) malonic acid	2.34 *17.80*	-	-	-
Methyl dehydroabietate	-	1.11 *20.20*	-	-
1-(*p*-Cumenyl)adamantane	-	1.84 *20.39*	-	-

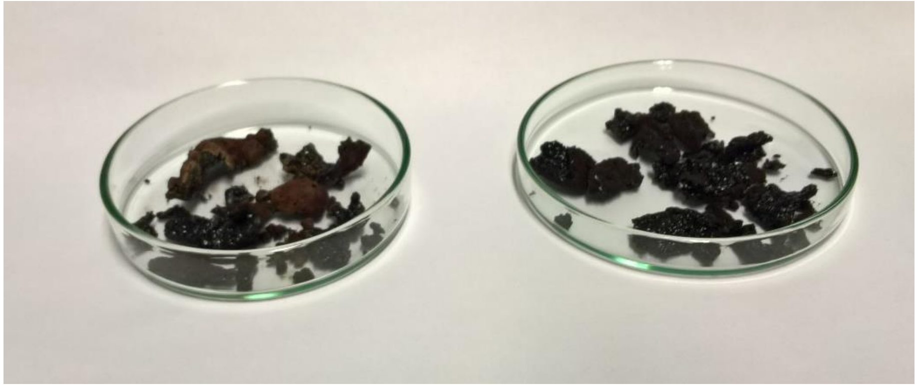

Figure S1. Materials after mild heat treatment at ~175 °C: lignin-decane (left), lignin-TOFA (right).

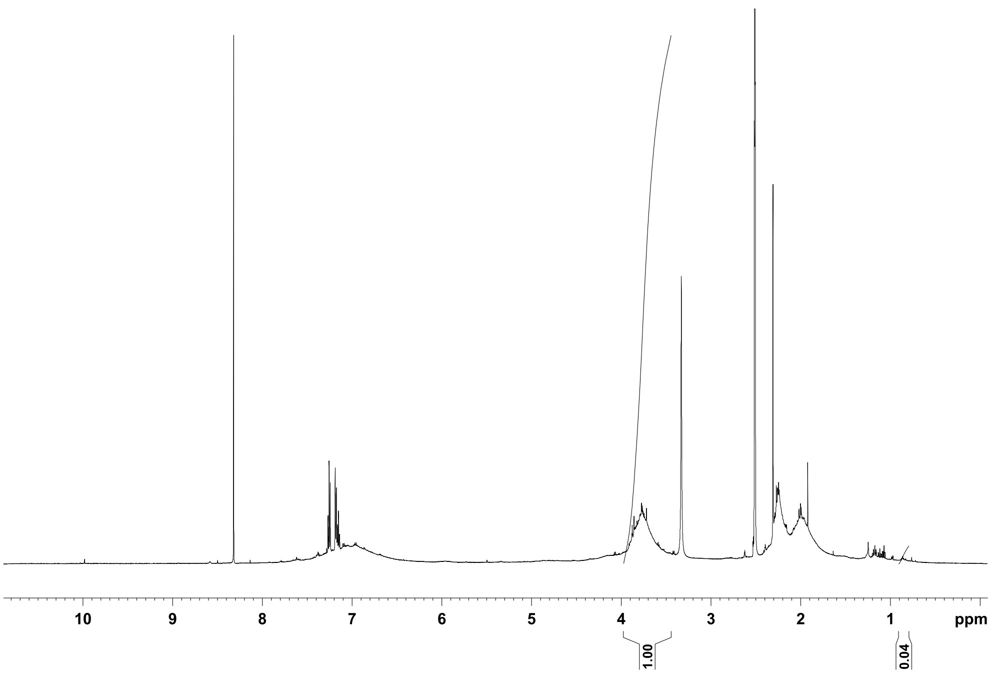

Figure S2. ^{1}H NMR spectrum of acetylated lignin before heat treatment. The integral value of 0.04 at ~0.9 ppm (Fω CH_3 of fatty acid) is related to the value of 1 at ~3.8 ppm (Ar-OCH_3 of a lignin unit and overlapping signals related to lignin side chain).

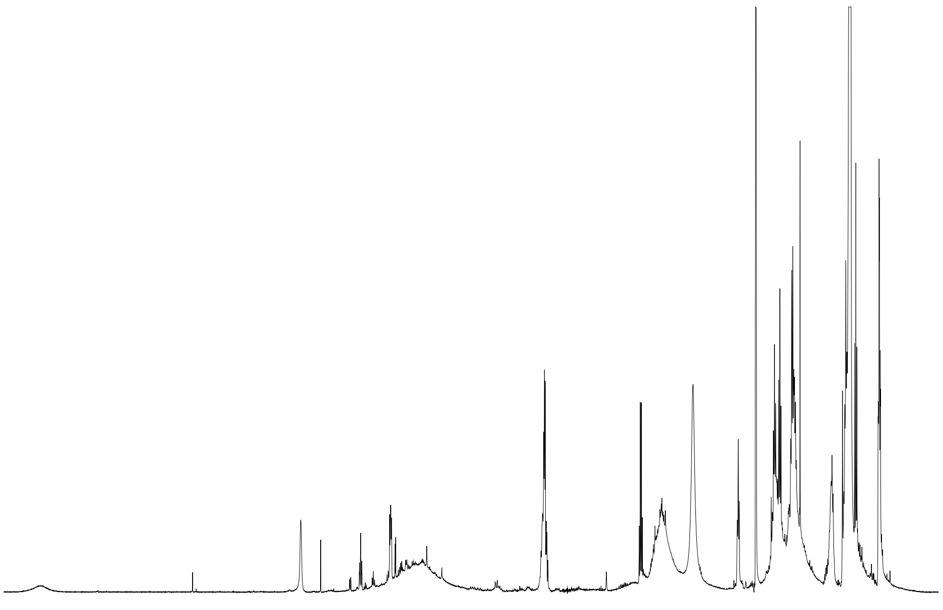

Figure S3. ^{1}H NMR spectrum of acetylated mixture of lignin and TOFA without heat treatment.

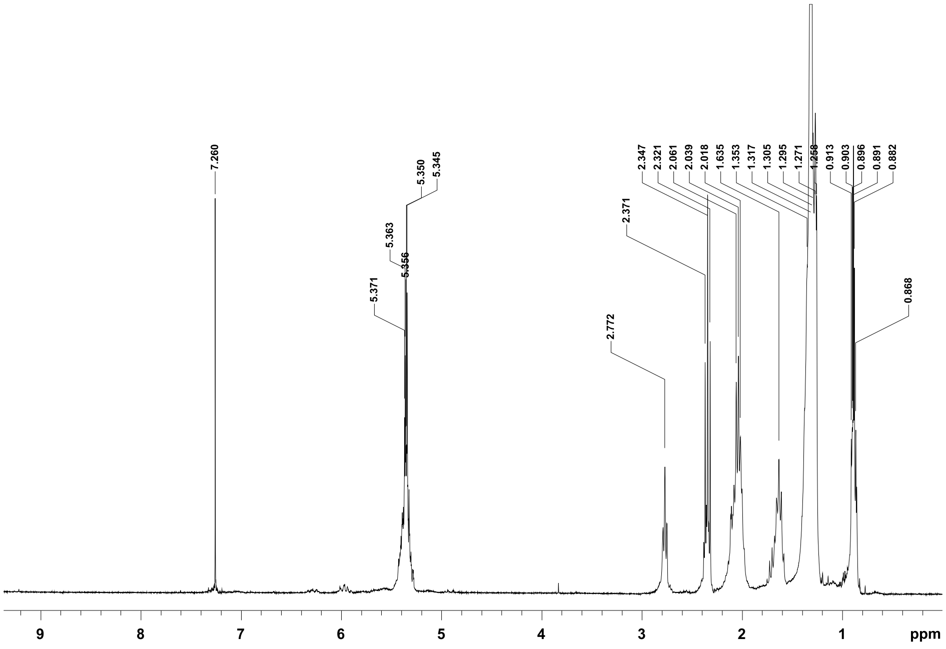

Figure S4. ^{1}H NMR spectrum of TOFA before heat treatment.

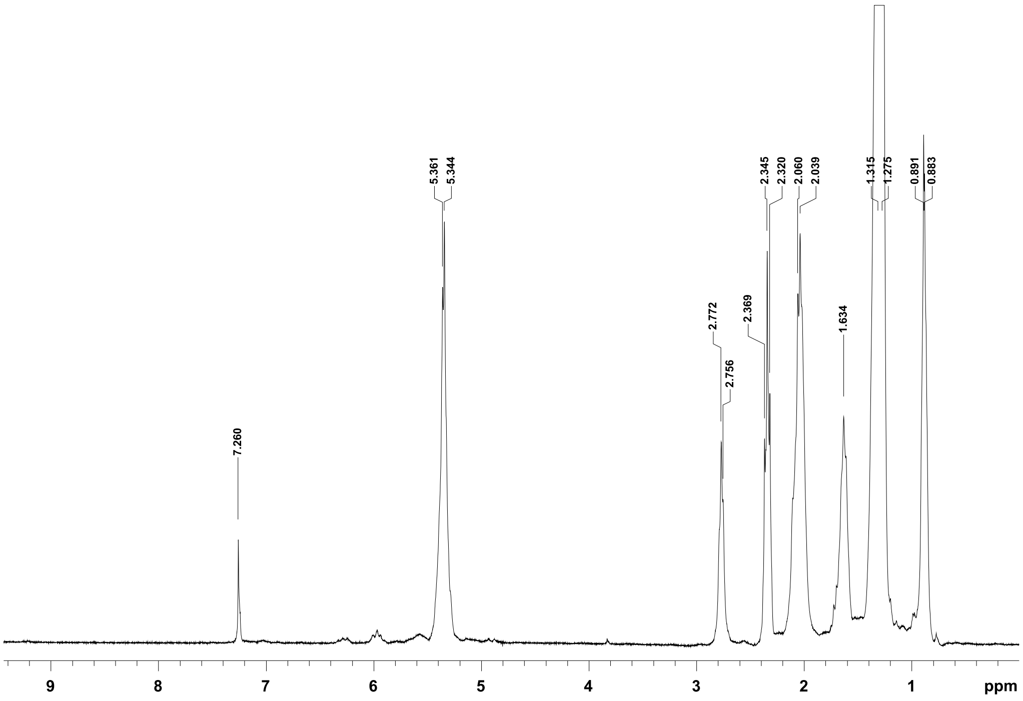

Figure S5. ^{1}H NMR spectrum of TOFA after heat treatment with lignin.

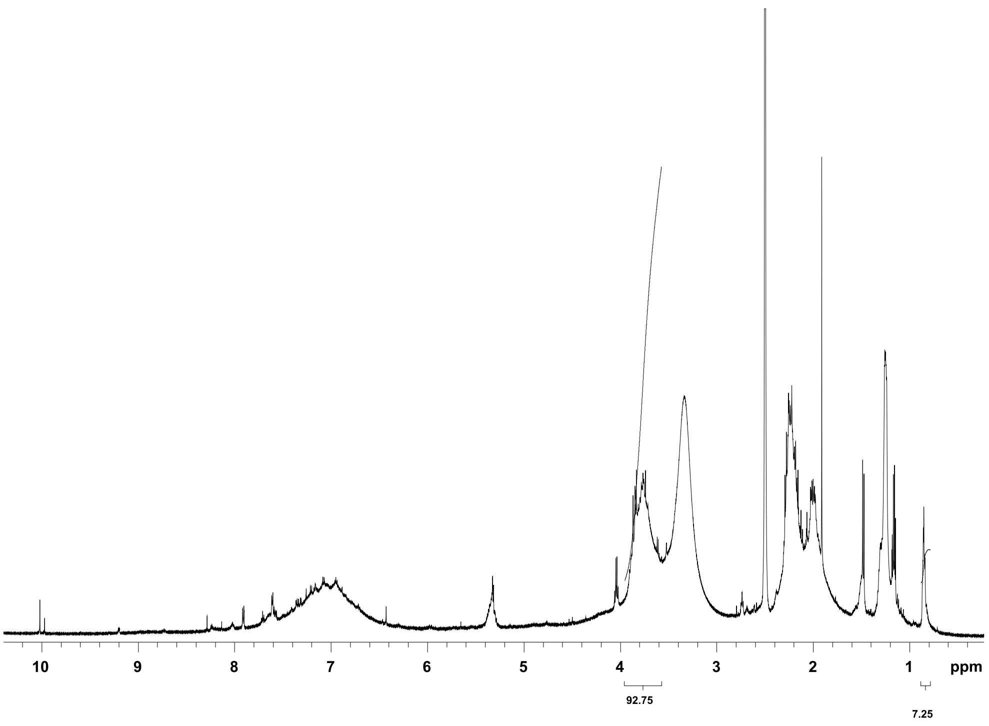

Figure S6. ^{1}H NMR spectrum of lignin-TOFA polymer after heat treatment. The sum of the presented integrals is 100.

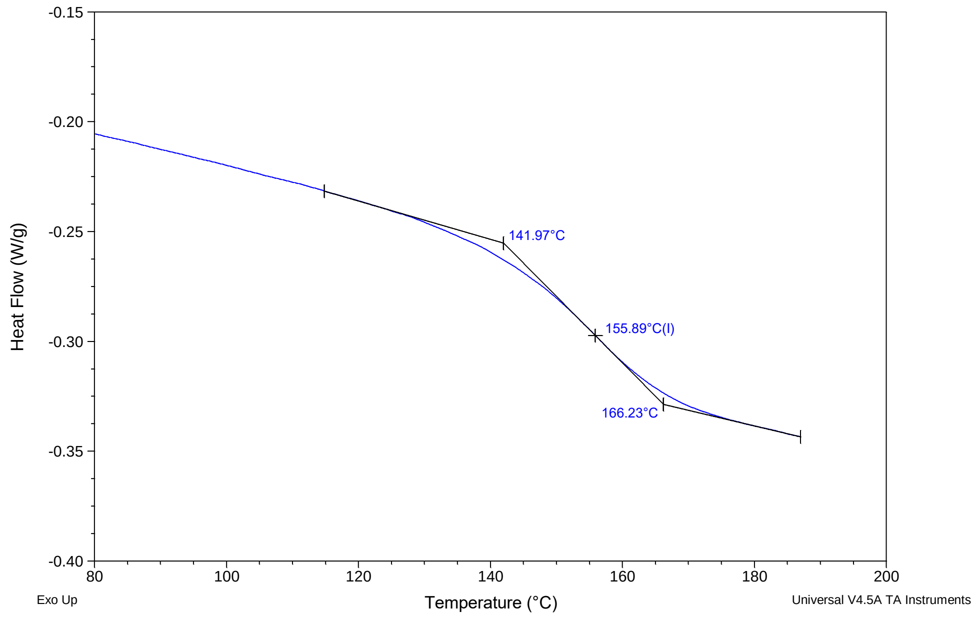

Figure S7. Differential scanning calorimetry (DSC) curve of starting kraft lignin.

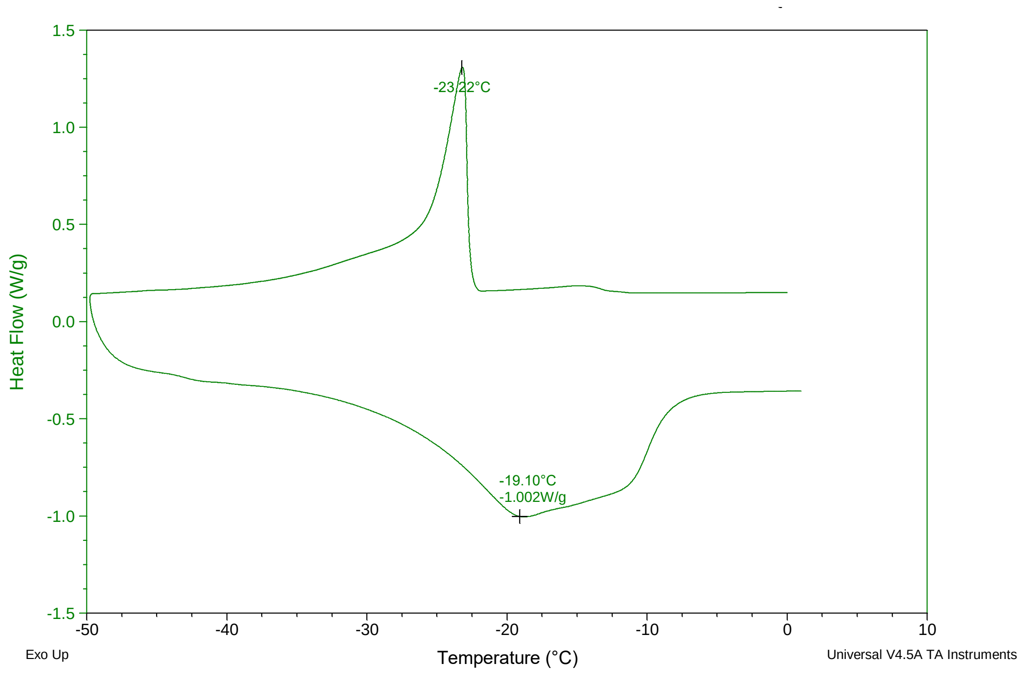

Figure S8. DSC curve of tall oil fatty acids (TOFA).

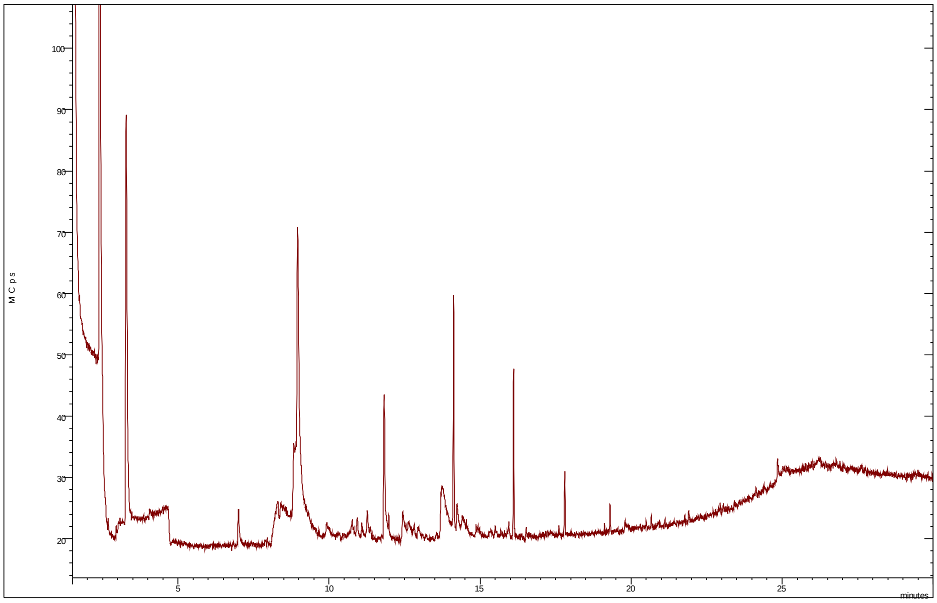

Figure S9. Pyrogram of starting lignin from Pyr-GC/MS analysis at 200 °C.

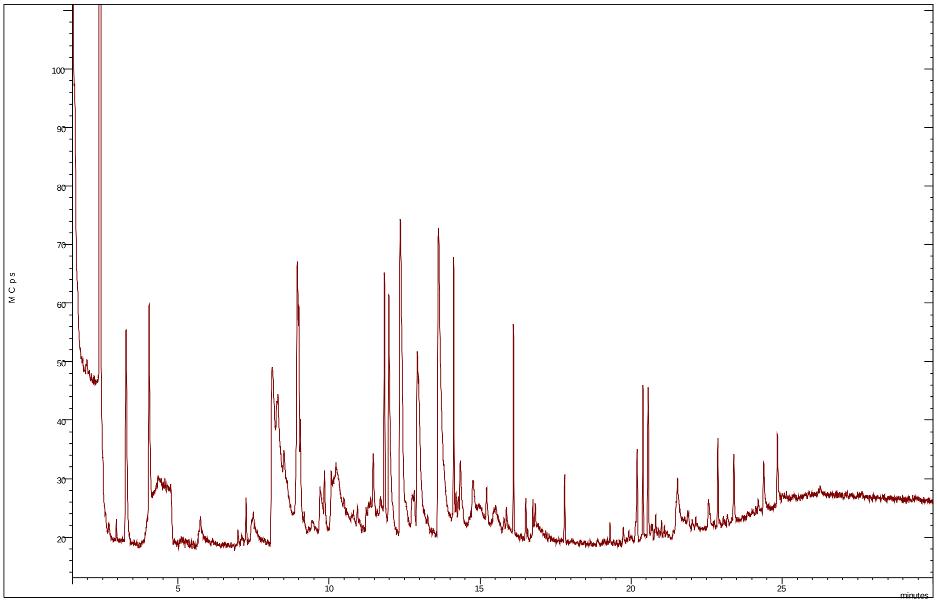

Figure S10. Pyrogram of lignin-TOFA mixture from Pyr-GC/MS analysis at 200 °C.

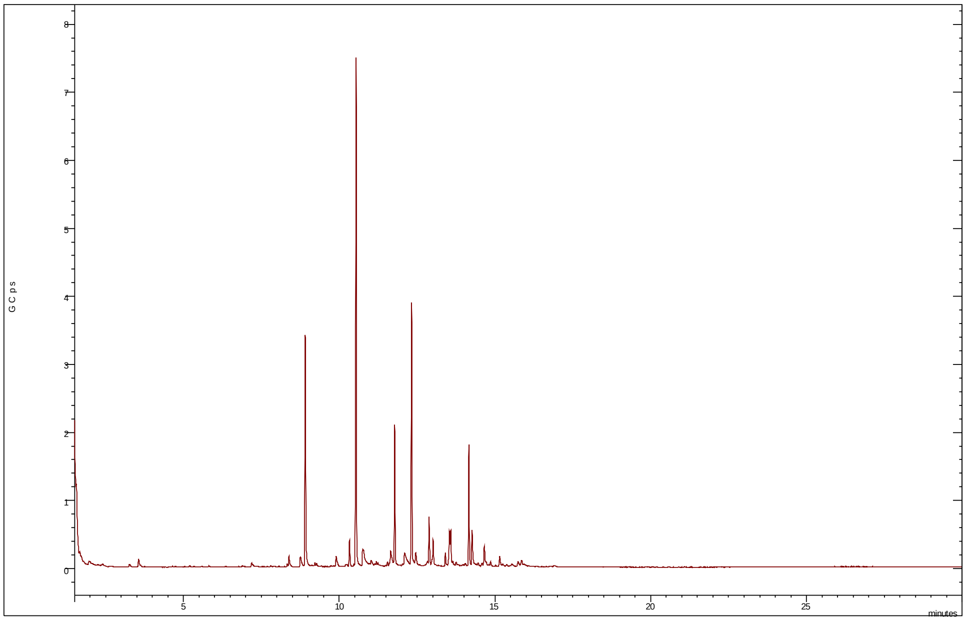

Figure S11. Pyrogram of starting lignin from Pyr-GC/MS analysis at 580 °C.

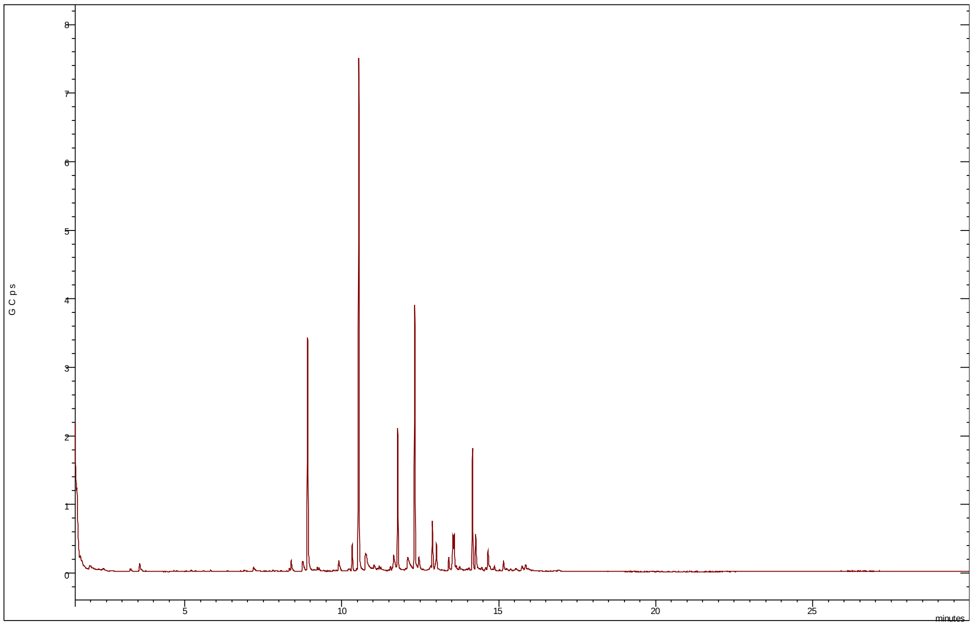

Figure S12. Pyrogram of lignin-TOFA mixture from Pyr-GC/MS analysis at 580 °C.

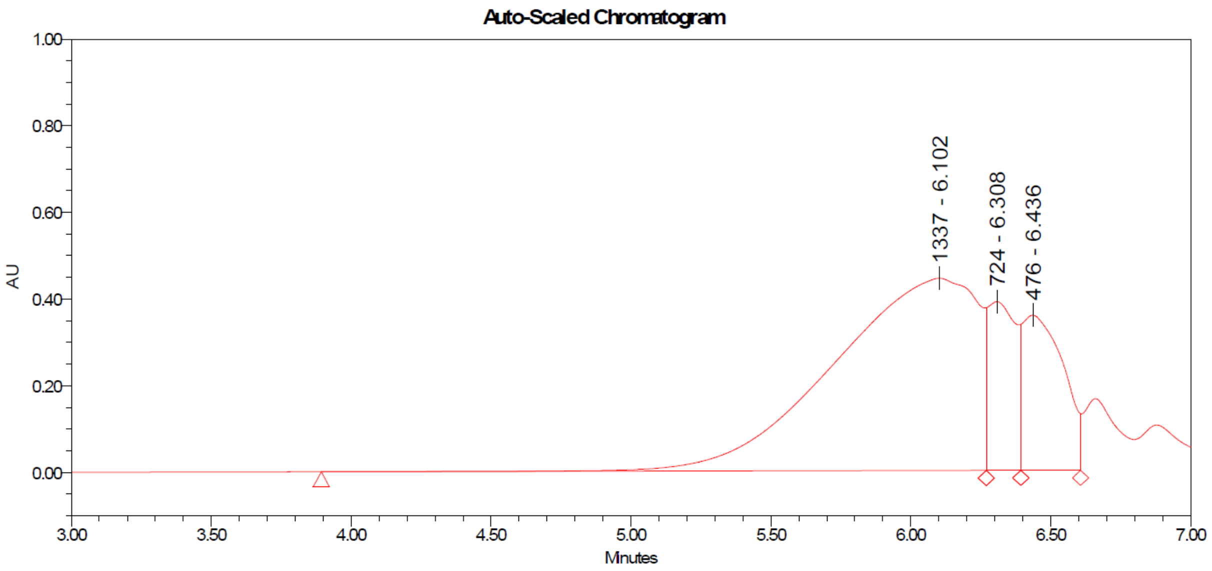

Figure S13. Size exclusion chromatography (SEC) curve of starting kraft lignin, which was acetylated in the presence of TOFA.

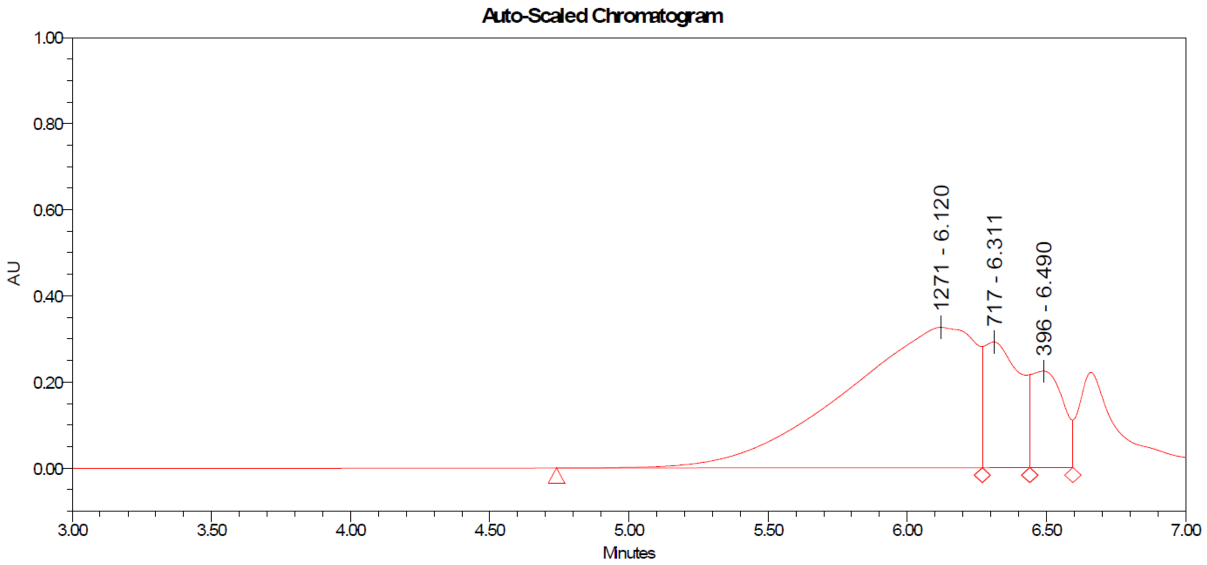

Figure S14. Size exclusion chromatography (SEC) curve of lignin-TOFA (acetylated).
